# Efficacy and safety of nafamostat mesylate versus heparin anticoagulation in adult kidney disease patients using continuous renal replacement therapy: a systematic review and meta-analysis

**DOI:** 10.3389/fmed.2026.1713412

**Published:** 2026-02-17

**Authors:** Yao Wang, Qiuhong He, Dan Wen, Rong Xu, Xiao Yu, Lingning Zhao

**Affiliations:** Intensive Care Unit, Mianyang Central Hospital, School of Medicine, University of Electronic Science and Technology of China, Mianyang, Sichuan, China

**Keywords:** anticoagulation, continuous renal replacement therapy, heparin, meta-analysis, nafamostat mesylate

## Abstract

**Background:**

Anticoagulation is essential during continuous renal replacement therapy (CRRT) for acute kidney injury to maintain circuit patency and balance bleeding risks.

**Objective:**

Systematically compare the anticoagulant efficacy and safety of nafamostat mesylate (NM) versus heparin in CRRT.

**Methods:**

We searched China National Knowledge Infrastructure, Wanfang Database, China Biology Medicine, PubMed, Embase, Cochrane Library, and Web of Science up to June 30, 2025 for randomized or non-randomized controlled trials. A meta-analysis was performed using RevMan version 5.4 software.

**Results:**

Seven retrospective cohort studies comprising were included. No significant differences between the two groups regarding filter lifespan [MD = -1.05; 95% CI (-5.92, 3.83); *P* = 0.67] and anticoagulation efficacy [OR = 2.64; 95% CI (0.41, 17.11); *P* = 0.31] were shown. No difference in the risk of bleeding events was shown [OR = 0.57; 95% CI (90.27, 1.21); *P* = 0.14]. The length of hospital stay in the NM group was significantly shortened [MD = –3.43; 95% CI (–5.53 to –1.33); *P* = 0.001]. NM showed significantly greater reduction in thrombin time (TT) compared to heparin [MD = –3.44; 95% CI (–5.33, –1.56); *P* = 0.0003), while no significant differences were observed in activated partial thromboplastin time (APTT) [MD = –5.35; 95% CI (–16.41, 5.72); *P* = 0.34) or international normalized ratio (INR) [MD = –0.46; 95% CI (–1.12, 0.20); *P* = 0.17].

**Conclusion:**

NM demonstrates similar filter lifespan and bleeding safety to heparin in CRRT. NM may shorten hospital stay and differentially affects coagulation indicators, supporting its use in individualized anticoagulation. But because of poor evidence, the conclusions must be interpreted with caution.

**Systematic review registration:**

https://www.crd.york.ac.uk/prospero/, identifier CRD420251077749.

## 1 Background

In the intensive care unit, up to 50% of patients develop acute kidney injury, which has a poor clinical prognosis (1). However, early detection and treatment can help restore kidney function. Continuous renal replacement therapy (CRRT) has been widely used for critically ill patients with acute kidney injury (2). Through a slow and isotonic blood purification process—during which electrolyte imbalances are corrected and volume overload prevented—CRRT can effectively eliminate inflammatory mediators (3). In so doing, this therapy plays a unique role in maintaining homeostasis of the internal environment. Premature filter failure is precipitated when the contact coagulation cascade is activated via blood–material interactions in extracorporeal circuits, resulting in rates of therapy discontinuation that exceed 34% (4). Frequent clotting events shorten the duration of treatment, increase healthcare costs and staff workload, and lead to elevated blood loss and transfusion requirements in patients (5).

Unfractionated heparin (UFH) is the most widely used anticoagulant. By enhancing antithrombin III activity, its use potently inhibits coagulation factors IIa and Xa, enabling rapid and reversible anticoagulation to be achieved (6). Beyond inhibiting the coagulation cascade, unfractionated heparin exhibits anti-inflammatory, immunomodulatory, and endothelium-protective functions (7). However, therapeutic dosing of heparin elevates bleeding risk, with an incidence of hemorrhagic complications of up to 40% being reported in the literature (8). This concern is particularly pronounced in patients with a high risk of bleeding at baseline (9).

Nafamostat mesylate (NM) exhibits an ultrashort half-life of 8 min and has specific inhibitory properties against coagulation factors IIa, VIIa, and Xa. In Japan, NM is utilized in 85% of cases of anticoagulation during CRRT (10). Its anti-inflammatory effect, achieved through inhibiting protease-activated receptor-1, provides additional benefits for patients with sepsis (11). The conclusions yielded by current studies regarding the safety and efficacy of NM are inconsistent: Some studies have demonstrated that NM significantly prolongs filter lifespan and reduces clotting rates (12). However, studies suggest that its benefits may be partially offset by potential adverse effects such as hyperkalemia and allergic reactions (13, 14).

Treatment guidelines recommend both NM and heparin regimens for anticoagulation during CRRT in patients who are critically ill (9). Consequently, this study aimed, through meta-analysis, to systematically evaluate the anticoagulant efficacy, safety, and adverse complications of administering NM versus heparin to patients who underwent CRRT.

## 2 Methods

The review and analysis were conducted in accordance with the PRISMA Preferred Reporting Items for Systematic reviews and Meta-Analyses guidelines (15). Our research plan was registered in PROSPERO (registration number CRD420251077749).

### 2.1 Inclusion and exclusion criteria

The inclusion criteria were formulated according to the patient population, intervention, comparison, and outcome (PICO) framework. The inclusion criteria were: (1) a study design compliant with those of randomized controlled trials, prospective cohort studies, or retrospective cohort studies; (2) interventions involving a direct comparison of NM versus unfractionated heparin for anticoagulation in CRRT; (3) study participants who were adult patients (aged ≥ 18 years) who met the Kidney Disease: Improving Global Outcomes (KDIGO)2012 criteria for initiating CRRT; and (4) outcome measures wherein at least one predefined outcome (including filter lifespan, bleeding events, coagulation parameters, anticoagulation efficacy, or length of hospital stay) was reported. Studies in which anticoagulation regimens that employed no anticoagulation, citrate anticoagulation, or argatroban anticoagulation, or those with non-extractable outcomes data or loss to follow-up of > 20% were excluded. Anticoagulation efficacy was defined as the percentage of patients exhibiting dialyzer coagulation graded as 0 or I. Coagulation severity was assessed according to the following criteria (16):

Grade 0: No coagulation or only a few clotted fibers.
Grade I: Less than 10% clotted fibers or the presence of small fiber bundles with clotting.
Grade II: Less than 50% clotted fibers or significant coagulation.
Grade III: More than 50% clotted fibers, a substantial rise in venous pressure, or the necessity for dialyzer replacement.

At the end of hemodialysis, the coagulation status within the dialyzer was examined. The anticoagulation efficacy rate was then calculated as the percentage of patients with coagulation graded as 0 or I, using the following formula: Anticoagulation efficacy rate = (Number of patients with Grade 0 or I coagulation/Total number of patients assessed) × 100%. Bleeding events were defined as instances of overt bleeding, a requirement for transfusion, or a reduction in hemoglobin exceeding 2 g/Dl (17).

### 2.2 Search strategy

A structured search strategy was employed to retrieve relevant literature from the following databases: China National Knowledge Infrastructure, Wanfang Database, China Biology Medicine, PubMed, Embase, Cochrane Library, and Web of Science. The timeframe of the search extended from the inception of each database through June 30, 2025. No language restrictions were applied to ensure broad coverage of both English and Chinese-language publications. Keywords included “nafamostat mesylate,” “heparin,” and “continuous renal replacement therapy.” Combining professional terminology with synonym expansion, the following search term combinations were constructed:

1. (“continuous renal replacement therapy”[tiab] OR “CRRT”[tiab] OR “continuous venovenous hemofiltration”[tiab] OR “CVVH”[tiab] OR “continuous hemodiafiltration”[tiab] OR “CHDF”[tiab]) OR (“renal replacement therapy”[Mesh] OR “hemofiltration”[Mesh])
2. (“nafamostat”[tiab] OR “nafamostat mesilate”[tiab])
3. (“heparin”[tiab] OR “unfractionated heparin”[tiab] OR “UFH”[tiab]) OR (“heparin”[Mesh])
4. Combinations 1 AND 2 AND 3

### 2.3 Literature screening and data extraction

The retrieved documents were imported into NoteExpress V4.0 (AegeanSoftware, Beijing, China). After removing duplicates, two reviewers trained in evidence-based methodology independently screened all the records by reviewing titles and abstracts as preliminary assessment for inclusion. After the initial screening, they read the full text of the articles that remained. The reviewers extracted data from the included studies, encompassing the first author’s name, year of publication, country, study design, sample size, patient characteristics, CRRT modality, anticoagulation regimen, and outcome measures.

### 2.4 Quality evaluation

Two reviewers independently assessed methodological quality using the Cochrane Risk of Bias tool (RoB 1.0) from the Cochrane Handbook for Systematic Reviews (Version 5.1.0) (18) as well as the Newcastle–Ottawa Scale (19). The Cochrane Handbook for Systematic Reviews focuses on seven critical domains: randomization, allocation concealment, blinding of participants and personnel, blinding of outcome assessment, incomplete outcome data, selective reporting, and other sources of bias. A three-tier classification system of *low risk of bias–high risk of bias–unclear* for item-by-item assessment was adopted. Appraisal of the methodological quality of the included cohort studies was performed using the ?Newcastle–Ottawa Scale, a validated tool used to evaluate eight domains across three core dimensions. Quality was assessed across three domains: selection of subjects, comparability of cohorts, and outcome assessment. Studies were classified as high (score, 6–8 points), moderate (4–5 points), or low (≤ 3 points) quality.

### 2.5 Data extraction

The quality of the literature was independently assessed by two reviewers trained in systematic analysis. Standardized operating procedures were implemented in the evaluation process, utilizing a two-way independent review system with methods for cross-verification and adjudication of discrepancies to minimize subjective bias; thereby, the reliability and reproducibility of the evaluation outcomes were ensured. When two reviewers disagreed on the assessment of a specific item in a study, they first resolved discrepancies through reexamining the original literature for discussion. If consensus remained unattainable, a third senior reviewer with certification in conducting Cochrane systematic reviews was engaged for a three-way review, with the final consensus serving as the definitive assessment of risk of bias for the relevant study item. All evaluation results were documented in predesigned standardized forms and subsequently incorporated into evidence synthesis after the accuracy was confirmed through two-way independent verification.

### 2.6 Statistical methods

The meta-analysis was performed using Review Manager 5.4 software and STATA statistical software (version 18.0) on data extracted from the literature, with the odds ratio (OR) and 95% confidence interval (CI) as effect measures. When heterogeneity testing indicated an *I*^2^ value of ≤ 50% and *P*-value of ≥ 0.1, a fixed-effect model was employed to calculate the pooled OR and 95% CI. An I^2^ value of > 50% and *P*-value of < 0.1 indicated significant heterogeneity. In such instances, sources were investigated via sensitivity or subgroup analyses. If homogeneity remained unattainable, the random effects model was applied for statistical synthesis. P < 0.05 indicated that the difference was statistically significant. We assessed the potential publication bias of the meta-analysis by using Egger’s tests.

## 3 Results

### 3.1 Literature search

The initial database retrieval identified 291 relevant studies. After removing duplicates, 185 studies were retained for initial screening. Through title and abstract screening, 58 studies were selected for full-text retrieval. After comprehensive assessment of the full text, seven studies were ultimately included in the meta-analysis (16, 17, 20–24). The literature screening process is detailed in Supplementary material 1.

### 3.2 Basic characteristics and quality of the included literature

The characteristics of the included literature are shown in Table 1. A total of seven studies were included in this research for a meta-analysis, all of which were cohort studies (16, 17, 20–24). The studies originated from East Asia: four from China (16, 21, 23, 24), two from Japan (20, 22), and one from South Korea (17). The patient population primarily consisted of adult patients who were critically ill, in intensive care units, and who required CRRT. The dosing regimens in the NM group comprised both fixed (10–50 mg/h) and weight-adjusted (0.1–0.5 mg/kg/h) strategies. In the heparin group, heparin was mainly administered, with the dosage ranging from 1 to 20 U/kg/h. All the included studies reported filter clotting and or longevity-related outcomes. Four studies (17, 22–24) reported hemorrhagic complications that were attributable to the therapy. Control for confounding and completeness of follow-up represent pervasive methodological limitations in cohort studies. The results of the quality appraisal of all the studies included are presented in Table 2.

**TABLE 1:** General characteristics of included literature.

Research	Research type	Nationality	Sample size (NM vs. heparin)	Patient characteristic	CRRT mode	Anticoagulation method	Primary outcome indicator
Kameda et al.	Cohort study	Japan	129 vs. 157	Adult patients requiring CRRT in ICU	CRRT	NM 10 mg/h vs. heparin 400 U/h	Filter life, hospital stay, mortality rate, mechanical ventilation time, inflammatory biomarkers
Liu and Li	Cohort study	China	33 vs. 32	End-stage renal failure patients on hemodialysis	CVVH	NM 20-50 mg/h vs. heparin5-10 mg/h	Clinical efficacy, anticoagulation effective rate (filter coagulation classification), coagulation indicators
Hwang et al.	Cohort study	Korea	25 vs. 56	Adult patients requiring CRRT in ICU	CVVH	NM 10-30 mg/h vs. heparin 1-20 U/kg/h	Filter life, coagulation indicators, bleeding events, survival rate
Makino et al.	Cohort study	Japan	76 vs. 25	Adult patients requiring CRRT in ICU	CRRT	NM 20 mg/h vs. heparin 400 IU/h	Filter lifespan, bleeding events
Miao and Chen	Cohort study	China	50 vs. 48	Patients with AKI due to sepsis	CVVH	NM 0.4 mg/kg/h vs. heparin 5-15 U/kg/h	Clinical efficacy, length of stay in the ICU, APACHE II score, immune indicators, renal function indicators, bleeding events, hyperkalemia
Zhan et al.	Cohort study	China	40 vs. 40	Patients with acute kidney injury (AKI)	CRRT	NM 0.4 mg/kg/h vs. heparin 3-15 U/kg/h	Filter lifespan, coagulation indicators, renal function indicators, bleeding events, hyperkalemia, hospital stay
Li et al.	Cohort study	China	44 vs. 42	Patients with CRRT (mainly with low platelet count)	CRRT	NM 0.1-0.5 mg/(kg/h) vs. heparin 5-15 U/(kg/h)	Anticoagulation effective rate (filter coagulation classification), coagulation indicators, and blood biochemical indicators

NM, Nafamostat mesylate; CRRT, Continuous renal replacement therapy; AKI, acute kidney injury; ICU, intensive care unit; RCT; randomized controlled trials; CVVH, continuous veno-venous hemofiltration.

**TABLE 2:** Newcastle-Ottawa quality assessment scale results of cohort studies.

Study ID	Selection				Comparability	Outcome			Total (9*)
	Representativeness of the exposed cohort (*)	Selection of non-exposed cohort (*)	Ascertainment of exposure (*)	Demonstration that outcome of interest was not present at start of study (*)	Comparability of cohorts (**)	Assessment of outcome (*)	Length of follow up(*)	Adequacy of follow up (*)	
Miao and Chen	*		*	*	**	*		*	7
Hwang et al.	*		*	*	*	*		*	6
Kameda et al.	*		*	*	**	*		*	7
Liu and Li	*	*			*	*		*	5
Makino et al.	*	*	*	*	**		*	*	8
Li et al.	*	*	*		*		*	*	6
Zhan et al.	*	*	*		*	*		*	6

*, ** means getting one point.

### 3.3 Outcomes analysis

#### 3.3.1 Filter lifespan

**FIGURE 1:**
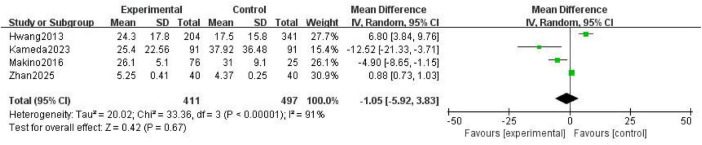
Forest plot comparing the filter lifespan between the nafamostat mesylate and heparin groups. CI, confidence interval; IV, intravenous; SD, standard deviation.

Four studies (17, 20, 22, 24) reported filter lifespan as an outcome measure. Significant heterogeneity was observed among them (*P* < 0.00001; *I*^2^ = 91%), and a random effects model was applied for analysis. The results showed that no statistically significant difference in the lifespan of the anticoagulant filter between the NM and heparin groups was present [mean difference (MD) = –1.05; 95% CI (–5.92, 3.83); *P* = 0.67) (Figure 1).

#### 3.3.2 Effectiveness in achieving anticoagulation

**FIGURE 2:**
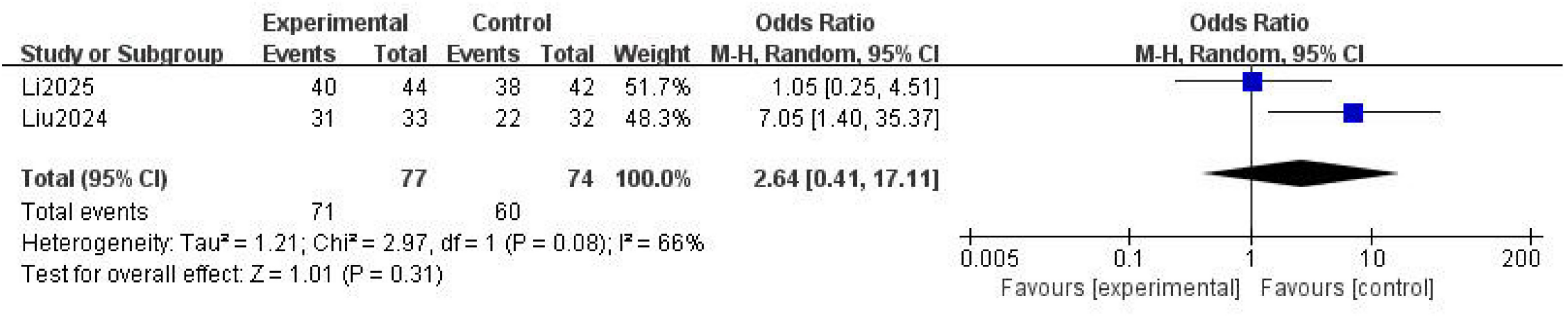
Forest plot comparing the effective rate of anticoagulation between the nafamostat mesylate and heparin groups. CI, confidence interval; M-H, Mantel-Haenszel.

Two studies (16, 21) reported effectiveness in achieving anticoagulation as an outcome measure. Moderate heterogeneity was observed among them (*P* = 0.08; *I*^2^ = 66%), and a random effects model was used for analysis: The results showed that no statistically significant difference in the effective rate of anticoagulation between the NM and the heparin groups was present (OR = 2.64; 95% CI [0.41, 17.11]; *P* = 0.31) (Figure 2).

#### 3.3.3 Bleeding events

**FIGURE 3:**
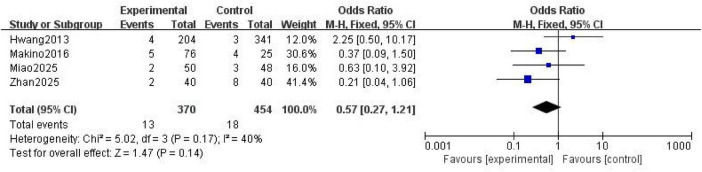
Forest plot comparing bleeding events between the nafamostat mesylate and heparin groups.

Four studies (17, 22–24) reported bleeding events as an outcome measure. No significant heterogeneity was observed among the included studies (*P* = 0.17; *I*^2^ = 40%), and a fixed effects model was applied for analysis. The results showed that no statistically significant difference in bleeding events between the NM and heparin groups was present (OR = 0.57; 95% CI [0.27, 1.21]; *P* = 0.14) (Figure 3).

#### 3.3.4 Length of hospital stay

**FIGURE 4:**
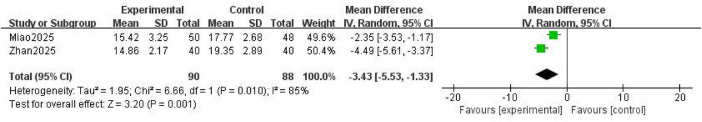
Forest plot comparing length of hospital stay between the nafamostat mesylate and heparin groups. CI, confidence interval; IV, intravenous; SD, standard deviation.

Two studies (23, 24) reported length of hospital stay as an outcome measure. Significant heterogeneity was observed between the two (*P* = 0.01; *I*^2^ = 85%), and a random effects model was used for analysis. The results showed a significant difference in the length of hospital stay between the NM and heparin groups (MD = -3.43; 95% CI [-5.53, -1.33]; *P* = 0.001) (Figure 4).

#### 3.3.5 Coagulation indicators

**FIGURE 5:**
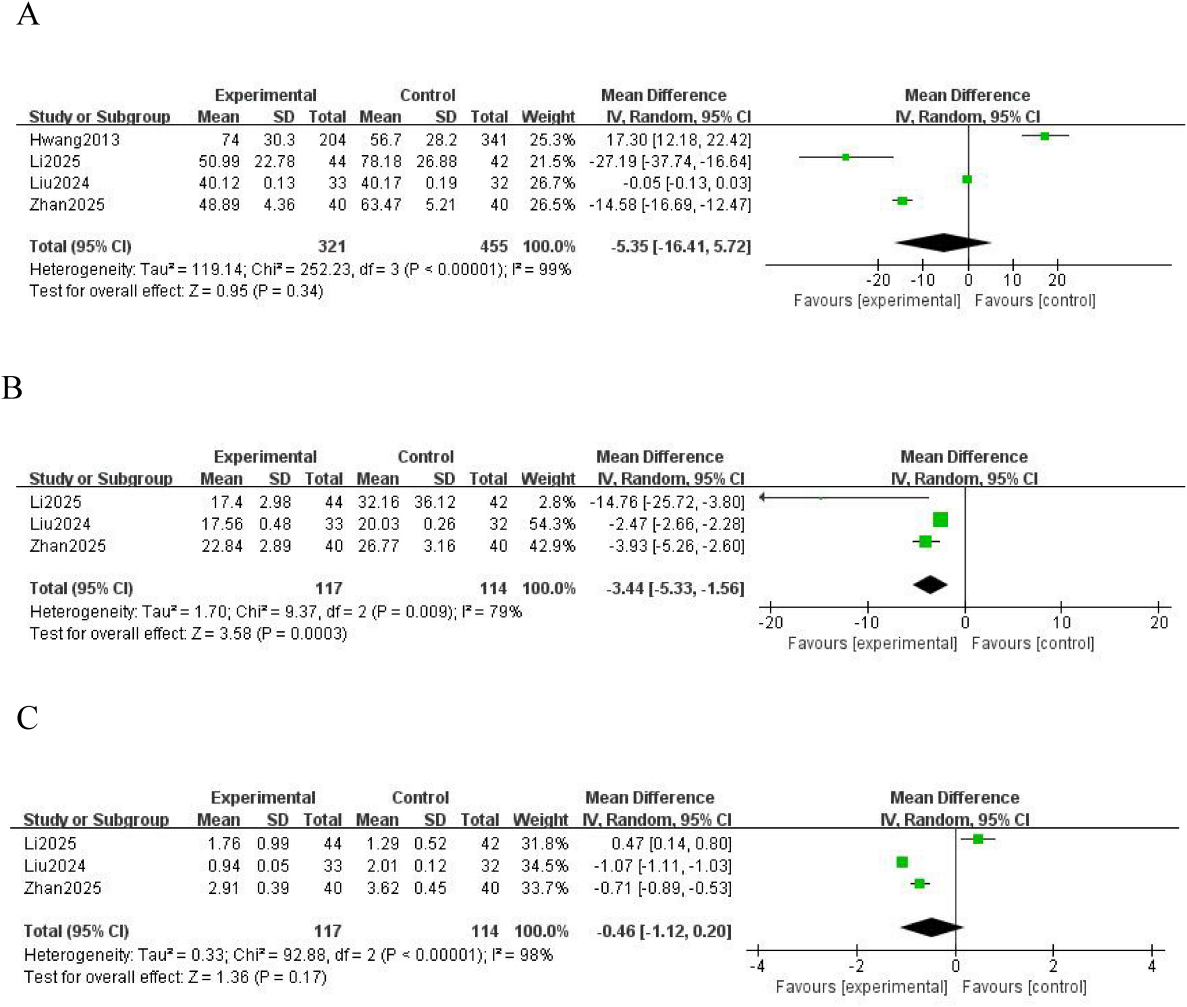
Forest plot comparing coagulation indicators between the nafamostat mesylate and heparin groups. **(A)** Forest plot of APTT. **(B)** Forest plot of TT. **(C)** Forest plot of INR. APTT, activated partial thromboplastin time; TT, thrombin time; INR, international normalized ratio; CI, confidence interval; IV, intravenous; SD, standard deviation.

Four studies (16, 17, 21, 24) reported the APTT as an outcome measure. Significant heterogeneity was observed among the included studies (*P* < 0.00001; *I*^2^ = 99%), and a random effects model was used for analysis. The results showed no statistically significant difference in the coagulation index, APTT, between the NM and heparin groups (MD = -5.35; 95% CI [-16.41, 5.72]; *P* = 0.34) (Figure 5A).

Three studies (16, 21, 24) reported the TT as an outcome measure. Significant heterogeneity was observed among the included studies (*P* = 0.009; *I*^2^ = 79%), and a random effects model was used for analysis. The results showed a statistically significant difference in the coagulation index, TT, between the NM and heparin groups (MD = -3.44; 95% CI [-5.33, -1.56]; *P* = 0.0003) (Figure 5B).

Three studies (16, 21, 24) reported the INR as an outcome measure. Significant heterogeneity was observed among these studies (*P* < 0.00001; *I*^2^ = 98%), and a random effects model was used for analysis. The results showed no statistically significant difference in the coagulation index, INR, between the NM and heparin groups (MD = -0.46; 95% CI [-1.12, 0.20]; *P* = 0.17) (Figure 5C).

#### 3.3.6 Subgroup analyses

To explore the substantial heterogeneity observed in several outcomes, pre-specified subgroup analyses were conducted based on anticoagulant dosing strategy. The subgroup analysis of filter lifespan revealed a statistically significant difference based on dosing strategy (*P* = 0.002). In studies employing a fixed-dose regimen, filter lifespan was significantly shorter in the NM group compared to the heparin group (MD = -7.62; 95% CI [-14.78, -0.47]; *P* = 0.04; Supplementary material 2). In contrast, no significant difference was found between groups in studies using a weight-adjusted dose (MD = 3.65; 95% CI [-2.14, 9.44]; *P* = 0.22; Supplementary material 2). The effect of NM on APTT was significantly modified by the dosing strategy (*P* = 0.008). The overall pooled result (MD = -5.35; 95% CI [-16.41, 5.72]; *P* = 0.34; Supplementary material 3a) masked divergent effects between the weight-adjusted and fixed-dose subgroups. An additional analysis by CRRT modality also indicated significant effect modification (*P* = 0.008; Supplementary material 3b). For INR (*P* = 0.11; Supplementary material 3c) and TT (*P* = 0.30; Supplementary material 3d), the subgroup analyses did not show statistically significant differences between weight-adjusted and fixed-dose subgroups, indicating that dosing strategy was not a major source of heterogeneity for these parameters.

#### 3.3.7 Sensitivity analysis and publication bias

Sensitivity analysis was performed using the one-study-removal approach to assess the stability of the effect size of all the studies combined. The results indicated that no single trial influenced the outcome measures, confirming the robustness of the findings. Egger’s regression analysis showed that no significant publication bias was found in the main outcome of filter lifespan (*P* = 0.780).

## 4 Discussion

In this meta-analysis, we pooled data from seven studies, providing the first systematic comparison of the safety and efficacy of using NM and heparin as the anticoagulant during CRRT. In the comparison of efficacy, no statistically significant difference was found in filter lifespan between the NM and heparin groups. Similarly, regarding safety, no statistically significant difference in the incidence of bleeding events between the two groups was shown (*P* > 0.05). For clinical outcomes, a significantly shorter length of hospital stay was shown in the NM group (*P* < 0.05). No statistically significant differences were shown for the coagulation monitoring indicators APTT and INR (*P* > 0.05), whereas a statistically significant difference was shown for the TT (*P* < 0.05).

The results of the present study showed no significant difference in filter lifespan between the NM or heparin groups (MD = -1.05; 95% CI [-5.92, 3.83]; *P* = 0.67). This contrasts with the conclusions reached in some studies (12) in which the filter lifespan was significantly prolonged when NM was used. Through in-depth analysis of sources of heterogeneity in the included studies, we identified diversity in dosing regimens as a key contributing factor. The dosage of NM covered a fixed dose of 10–50 mg/h and a weight-adjusted dose of 0.1–0.5 mg/kg/h, whereas the dosage of heparin ranged from 1 to 20 U/kg/h. NM selectively inhibits coagulation factors XIIa and XIa, and kallikreins, thereby blocking the intrinsic coagulation pathway. Heparin relies on Antithrombin III to inhibit coagulation factors XIIa and XIa (25, 26). Although their anticoagulation targets differ, both NM and heparin effectively delay the deposition of fibrin within the filter circuit. The cohort study conducted by Kameda et al. (20) involving 286 patients in the intensive care unit demonstrated a median filter lifespan of 38.2 h in the NM group versus 36.5 h in the heparin group (*P* = 0.78).

No statistically significant difference in the incidence of bleeding events was shown between the two groups (OR = 0.57; 95% CI [0.27, 1.21]; *P* = 0.14), providing critical evidence for selecting an anticoagulant for patients at high risk of bleeding. However, due to the limited number of studies reporting this result, it must be regarded as a preliminary conclusion. Also, the potential influence of publication bias on this non-differential outcome should be taken into consideration. The risk of bleeding associated with heparin correlates with its comprehensive suppression of the coagulation cascade, whereas the targeted inhibition exhibited by NM minimizes interference with physiological hemostasis (27). Additionally, the risk of heparin Induced thrombocytopenia (HIT) must be considered (28). In the cohort studied by Makino (22), two cases of suspected HIT were noted in the heparin group, which may partially explain the discrepancy in length of hospital stay. However, in this study, the administration of NM did not significantly reduce the number of bleeding events. This finding may be related to the following factors: Firstly, the proportion of patients at high risk of bleeding included in the studies was insufficient; Secondly, the dose adjustment of NM was not fully matched to individual patient variations. Notably, the utilization rate of NM for anticoagulation in CRRT in the Japanese guidelines reaches 85% (29); because NM has an ultra-short half-life, it is particularly suitable for use in clinical scenarios in which frequent interruption of anticoagulation therapy is required. Clinically, when administering NM, particular attention must be paid to the continuity of the administration route to avoid fluctuations in the anticoagulant effect due to interruption of infusion. Similarly, during administration of heparin therapy, vigilance against concurrent risks of anticoagulation failure and bleeding caused by ATIII depletion is required (30).

The length of hospital stay in the NM group was significantly shorter than that in the heparin group (MD = -3.43; 95% CI [-5.53, -1.33]; *P* = 0.001). However, this favorable result is based on only two studies, and its robustness should be interpreted with caution until confirmed by larger, prospective investigations. The mechanism underlying this finding may involve the multifaceted pharmacological effects of NM. Besides its anticoagulant action, NM inhibits protease-activated receptor-1, thereby reducing the release of inflammatory mediators such as interleukin 6 and tumor necrosis factor-alpha (31). The study conducted by Miao and Chen (23) confirmed that patients with sepsis-associated acute kidney injury who were treated with NM demonstrated a greater reduction in Sequential Organ Failure Assessment scores compared to those treated with heparin. This reduction accelerated the recovery of organ function and consequently shortened the length of hospital stay. However, vigilance is required against adverse reactions, such as hyperkalemia, that the administration of NM may induce (23). Zhan et al. (24) reported an incidence of hyperkalemia in the NM group of 12.5%. Clinical monitoring should include an increased frequency of electrolyte testing, especially in patients with renal insufficiency, while emphasizing control of the infusion rate and management of the electrolyte balance.

The APTT primarily reflects the functionality of the intrinsic coagulation pathway. Heparin significantly prolongs the APTT by potently inhibiting coagulation factors Xa and IIa through enhanced ATIII activity (32). NM inhibits intrinsic coagulation factors such as XIIa and Xa. However, its ultra-short half-life and regionally specific anticoagulation properties result in high local concentrations within the extracorporeal circuit, while rapid systemic clearance minimizes its impact on the systemic APTT (33). NM is rapidly metabolized by hepatic carboxylesterases, with approximately 80% of it being cleared by the liver, while dialysis devices can partially remove it (34). Therefore, the risk of systemic accumulation is low. Heparin is primarily cleared by the kidneys. In cases of renal insufficiency, the half-life of heparin is prolonged, resulting in greater fluctuations in the APTT. TT directly reflects the activity of coagulation factor IIa. NM potently inhibits thrombin, thereby significantly shortening the TT. Although heparin inhibits factor IIa, its action is dependent on ATIII and subject to interference from heparinase, PF4, thus limiting its impact on the TT (35). This property makes heparin particularly suitable for use in patients at high risk of bleeding. Li et al. (16) demonstrated that, in patients with platelet counts of < 50 × 10^9^/L, the rate of bleeding reached 14.3% in the heparin group, whereas it only reached 6.8% in the NM group. This indicates that, for clinical monitoring, priority should be given to the TT values when administering NM, but that the APTT should serve as the primary indicator when administering heparin therapy. Clinically, the frequency of monitoring coagulation and interpretation of these parameters must be adjusted based on the type of anticoagulant administered. The INR primarily reflects the extrinsic coagulation pathway. No significant difference in the INR was shown between the two groups, because neither NM nor heparin primarily targets this pathway. This indicates that the effect of NM on vitamin K is similar to that of heparin. However, the rapid inhibitory effect of NM on thrombin may interfere with global coagulation assessments such as thromboelastogram. Clinical staff must integrate the mechanism of action of the anticoagulant to comprehensively interpret coagulation reports.

In the subgroup analysis, we found that the anticoagulant dosing strategy served as a major effect modifier, particularly for filter lifespan and APTT. The inferior performance of NM under fixed-dose regimens suggests that a fixed, non-individualized dosing strategy may lead to under-anticoagulation, compromising filter longevity. This underscores the paramount importance of utilizing weight-adjusted dosing to optimize the efficacy of NM in CRRT, ensuring adequate anticoagulation while potentially minimizing adverse effects. Furthermore, the influence of CRRT modality on APTT highlights the complexity of comparing anticoagulation across different technical settings. These findings shift the focus from a simple drug-versus-drug comparison to a more nuanced understanding that includes protocol-specific factors, advocating for personalized anticoagulation management in critically ill patients undergoing CRRT.

### 4.1 Limitations

This study had the following limitations: First, significant heterogeneity was observed for several outcomes, which although explored through subgroup analyses, remains a consideration. Second, the generalizability of the findings may be limited as all included studies were conducted in East Asia; the applicability of the results to other populations with different genetic backgrounds, clinical practices, or risk profiles requires further investigation. Third, we explicitly acknowledge that all included studies were non-randomized in design.

While the results are consistent and provide valuable insights from real-world clinical practice, the conclusions must be interpreted with caution. Finally, the number of studies reporting key outcomes like hospital stay and bleeding events was relatively small, which may limit the robustness of those specific conclusions.

## 5 Conclusion

The findings of this meta-analysis demonstrated that NM and heparin provide comparable filter longevity and hemorrhagic safety profiles when used as anticoagulants during CRRT. NM exhibited the distinct advantages of significantly shortening length of hospital stay and optimizing regulation of coagulation parameters. In clinical practice, formulating tailored monitoring protocols necessitates the integration of individual patient characteristics with the pharmacological properties of the anticoagulant. A pressing need for more high-quality RCT research exists to establish a more robust evidence base for developing anticoagulation strategies in CRRT. Future large-scale studies, specifically powered to detect differences in patient-centered outcomes like hospital stay and bleeding events, and crucially, conducted in diverse geographic settings beyond East Asia, are essential to confirm the generalizability of our results and to establish NM’s role in a global context.

## Data Availability

The original contributions presented in this study are included in this article/Supplementary material, further inquiries can be directed to the corresponding author.
