# Supplementary figures and images for: Efficacy and safety of nafamostat mesylate versus heparin anticoagulation in adult kidney disease patients using continuous renal replacement therapy: a systematic review and meta-analysis
Source: Front Med (Lausanne). 2026 Feb 17;13:1713412. doi: 10.3389/fmed.2026.1713412 (PMC12953472; doi:10.3389/fmed.2026.1713412)

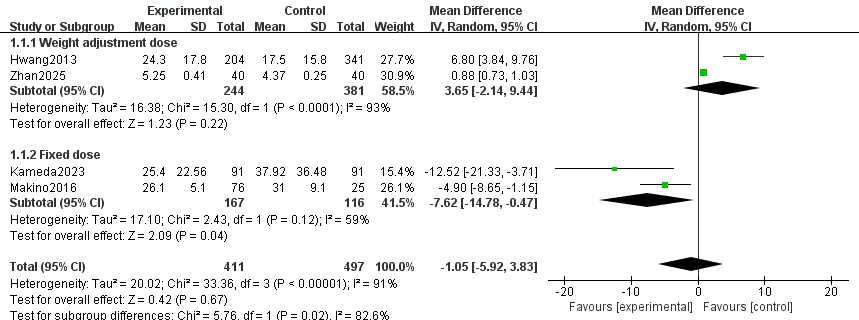


Supplementary materials 2. Forest plot of subgroup analysis of filter lifespan.

Supplement: Supplementary file 1 [file Data_Sheet_1.docx]
