# Supplementary material for: Efficacy and safety of nafamostat mesylate versus heparin anticoagulation in adult kidney disease patients using continuous renal replacement therapy: a systematic review and meta-analysis
Source: Front Med (Lausanne). 2026 Feb 17;13:1713412. doi: 10.3389/fmed.2026.1713412 (PMC12953472; doi:10.3389/fmed.2026.1713412)
Supplement: Supplementary file 2 [file Table_1.docx]

Records identified through databases search (n=291):

Pubmed:45, CINAHL:15, MEDLINE: 67, Cochrane Library: 4, Web of Science: 15, CNKI: 51, Wanfang: 62, SinoMed: 32

Duplicate removed (n=106)

**Identification**

Records excluded (n=127):

irrelevant topic (n=66)

irrelevant population (n=21)

case reports (n=23)

meta-analysis or systematic review (n=17)

Records screened by tittle and abstract after duplicates removed (n=185)

**Screening**

Reports excluded(n=51):

insufficient statistics for analysis (n=8)

inappropriate intervention (n=32) not reporting outcomes of interest (n=11)

)

Reports assessed for eligibility

(n=58)

Studies included in meta-analysis(n=7)

**Included**

Supplementary materials 1. PRISMA 2020 flow diagram.
