# Supplementary material for: Efficacy and safety of nafamostat mesylate versus heparin anticoagulation in adult kidney disease patients using continuous renal replacement therapy: a systematic review and meta-analysis
Source: Front Med (Lausanne). 2026 Feb 17;13:1713412. doi: 10.3389/fmed.2026.1713412 (PMC12953472; doi:10.3389/fmed.2026.1713412)
Supplement: Supplementary file 3 [file Table_2.docx]

a. Subgroup analysis based on dose for APTT


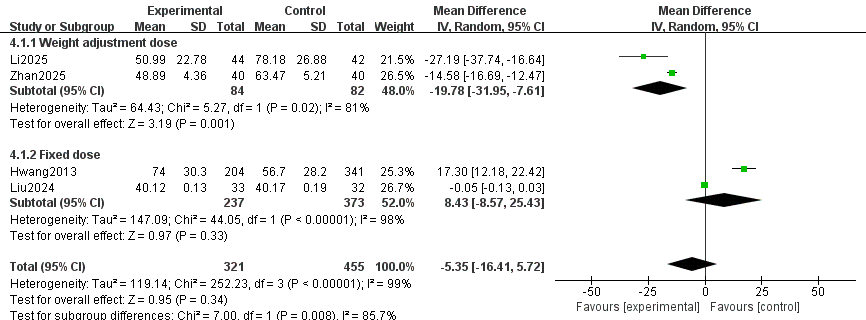


b. Subgroup analysis based on modality for APTT


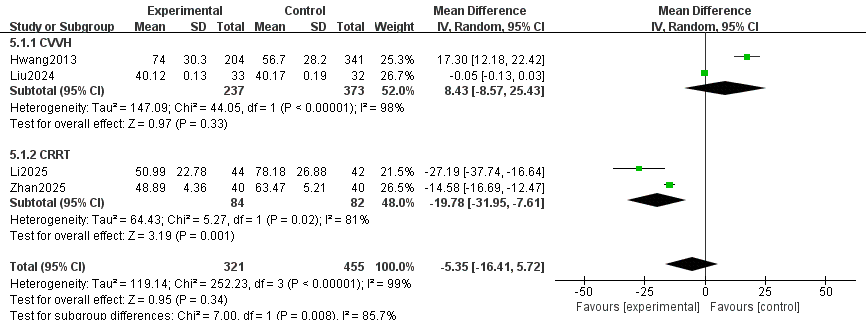


c. Subgroup analysis based on dose for INR


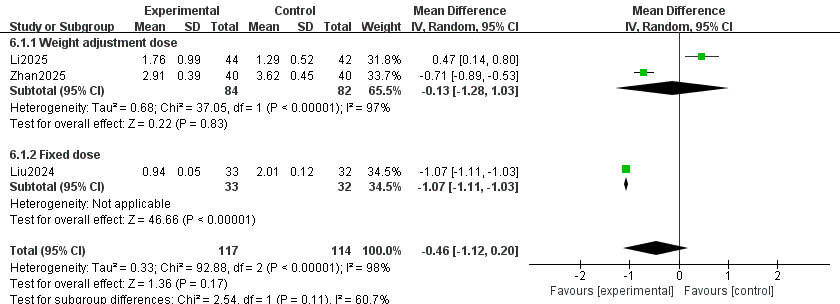


d. Subgroup analysis based on dose for TT


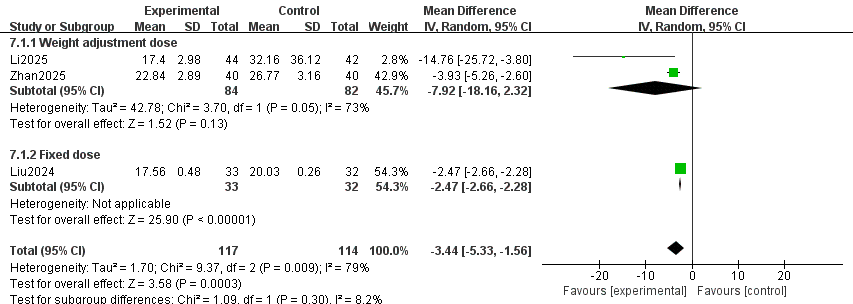


Supplementary materials 3. Forest plot of subgroup analysis of coagulation indicators.
